# Normal High HbA1c a Risk Factor for Abnormal Pain Threshold in the Japanese Population

**DOI:** 10.3389/fendo.2019.00651

**Published:** 2019-10-02

**Authors:** Chieko Itabashi, Hiroki Mizukami, Sho Osonoi, Kazuhisa Takahashi, Kazuhiro Kudo, Kanichiro Wada, Wataru Inaba, Guo Danyang, Chiaki Uchida, Satoko Umetsu, Akiko Igawa, Saori Ogasawara, Masaki Ryuzaki, Kouji Komeda, Yasuyuki Ishibashi, Soroku Yagihashi, Shigeyuki Nakaji

**Affiliations:** ^1^Department of Pathology and Molecular Medicine, Hirosaki University Graduate School of Medicine, Hirosaki, Japan; ^2^Department of Orthopedic Surgery, Hirosaki University Graduate School of Medicine, Hirosaki, Japan; ^3^Department of Gastrointestinal Surgery, Hirosaki University Graduate School of Medicine, Hirosaki, Japan; ^4^Department of Social Medicine, Hirosaki University Graduate School of Medicine, Hirosaki, Japan

**Keywords:** small fiber dysfunction, diabetic polyneuropathy, HbA1c, painful, small fiber assessment

## Abstract

**Purpose:** Small fiber dysfunction is common in subjects with diabetic polyneuropathy (DPN). It is unsettled, however, whether marginal glucose intolerance is implicated in the onset and progression of small fiber dysfunction. Herein, we explored the relationship between glycated hemoglobin levels (HbA1c) and pain sensation in the Japanese population.

**Methods:** A population-based study of 894 individuals (352 men, 542 women; average age 53.8 ± 0.5 years) and 55 subjects with impaired fasting glucose (IFG) in the 2017 Iwaki project were enrolled in this study. Individuals with diabetes were excluded. Relationships between pain threshold for intraepidermal electrical stimulation (P-IES) and parameters associated with metabolic syndrome were examined.

**Results:** P-IES was elevated with increasing of age in women but not in men. Average P-IES (mA) was increased in IFG subjects (*n* = 55, 0.20 ± 0.03) compared with normoglycemic/non-IFG individuals (*n* = 894, 0.15 ± 0.01) (*p* < 0.01). It was comparable between IFG and a group of normal high HbA1c (5.9–6.4%). Univariate linear regression analyses showed no influence of sex, triglyceride, or cholesterol on the value of P-IES. In contrast, there were significant correlations between P-IES and serum HbA1c level (ß = 0.120, *p* < 0.001) Adjustments for the multiple clinical measurements confirmed positive correlation of P-IES with HbA1c (ß = 0.077, *p* = 0.046).

**Conclusion:** Individuals with normal high HbA1c exhibited an elevated P-IES in a healthy Japanese population which may be useful for the screening of subclinical DPN.

## Introduction

Diabetic polyneuropathy (DPN) is the most common and earliest diabetic microvascular complication (1). Manifestations can include “positive” symptoms such as prickling, burning, and aching sensations, or “negative” symptoms, such as decreased sensation and numbness, all of which reduce patient's quality of life. There is currently no effective treatment approved globally.

DPN is a slowly progressive sensory predominant neuropathy characterized by a distal “dying back” type, in which nerve fibers are retrogradely injured from periphery (2). In this type of neuropathy, small fibers in the distal foot are often first to be affected. Symptoms of DPN can be apparent in prediabetes as well as in overt diabetes (3). Small fiber neuropathy (SFN) is the earliest form of DPN in patients with impaired glucose tolerance (IGT) who experience significant loss or dysfunction of small intraepidermal nerve fibers without large fiber defects (4). Patients with SFN can present with neuropathic pain in the feet, however, the condition can also be pain free, with absent or reduced pain and temperature sensation (5). Dysfunction or loss of small fibers is also observed in preclinical asymptomatic DPN. Therefore, asymptomatic preclinical DPN is difficult to distinguish from SFN without a large fiber evaluation. Although there is also some controversy as to whether prediabetic DPN really exists (6), new diagnostic tools such as corneal confocal microscopy confirm the reduction of small fiber density and length in IGT (7).

On the other hand, recent studies have revealed that the loss of small nerve fibers can be restored in pre-diabetic individuals with lifestyle interventions or in patients with overt diabetic neuropathy by pancreas transplantation (8). HbA1c, an indicator for diabetes diagnosis, is also a predictor of future type 2 diabetes (9, 10). Individuals with a normal high HbA1c value (6.0–6.4%) have been found to be at higher risk for the onset of type 2 diabetes than those with impaired fasting glucose (IFG) (9). It is unknown, however, whether normal high HbA1c is an indicator of small fiber dysfunction. Taking into account the fact that normal high HbA1c is a predictor of diabetes, it is also assumed to be a risk factor for small fiber dysfunction.

Currently, small fiber dysfunction in diabetes can be quantitatively evaluated by skin biopsy or corneal confocal microscopy (11, 12). However, non-invasive reproducible, easily repeatable, and quantifiable methods for clinical evaluation of small fiber function are still needed. A new electrode for intraepidermal electrical stimulation (IES) makes it possible to selectively stimulate cutaneous Aδ and C fibers, which convey pain and temperature sensation. Alterations in the response from those terminal epidermal small fibers could thus reflect the impaired pain sensation (13). In fact, there was a significant increase in the IES pain threshold (P-IES) in patients with DPN compared with those without DPN (14, 15). There are no reference normative values or ranges of variation regarding P-IES or clinical risk factors to worsen P-IES in a general non-diabetic healthy population.

In this study, we evaluated P-IES in a general Japanese population exhibiting normal fasting glycemia and HbA1c and obtained male and female average P-IES in each decade of life. We also explored the possibility of normal high HbA1c levels as P-IES screening indices for small fiber dysfunction.

## Materials and Methods

### Participant Demographics

**Figure 1:**
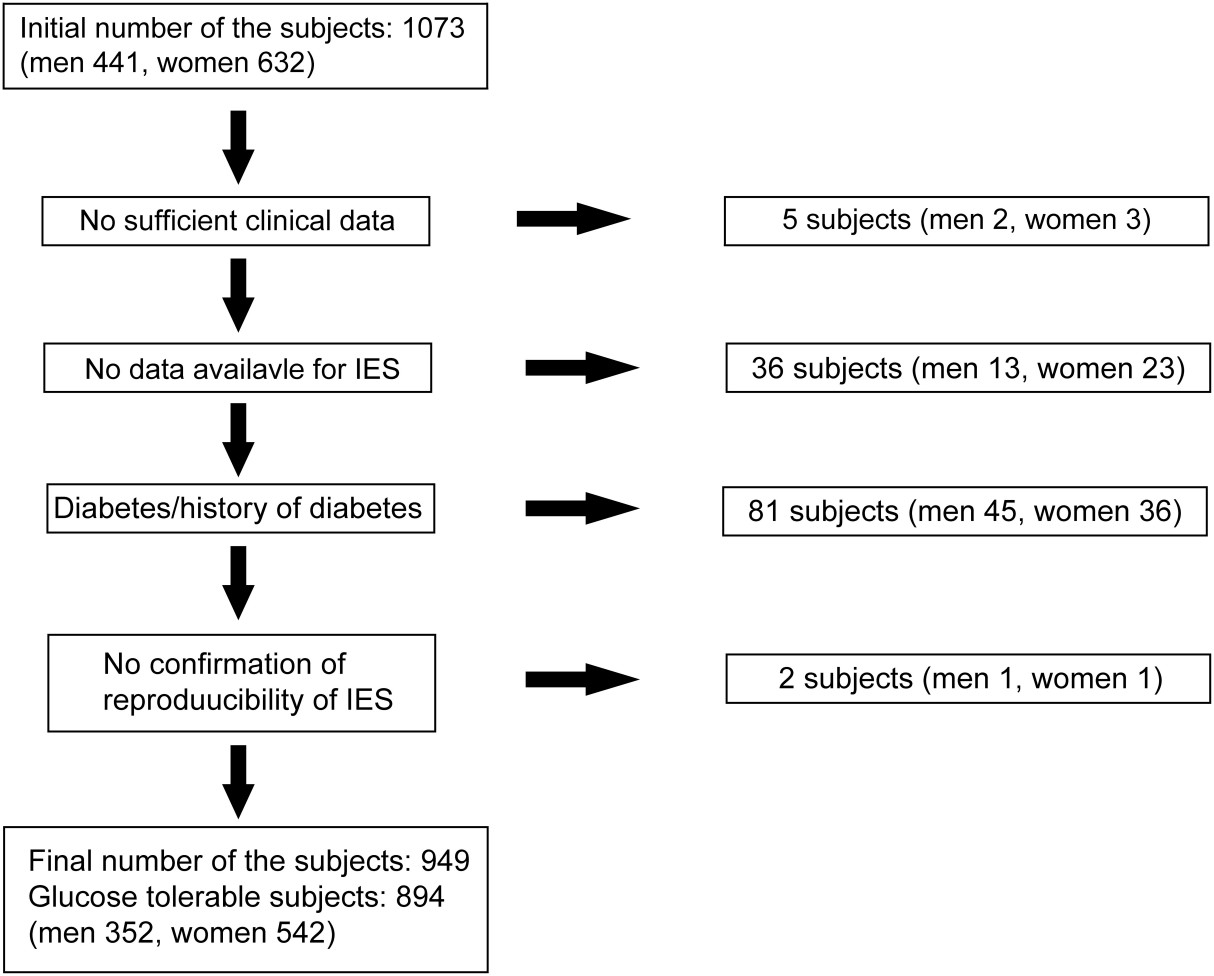
Subjects selection. Eight hundred ninety-four normoglycemic precipitants (352 men, 542 women) were finally examined out of 1,073 volunteers from the Iwaki study 2017 in this study.

We recruited fasting normoglycemic (Control) individuals and those who IFG without a history of diabetes who participated as volunteers in the Iwaki study, a health promotion study of Japanese individuals over 10 years of age. This project began in 2007, lasted 10 years, and aimed to prevent lifestyle-related diseases and prolong lifespans. This study was performed in accordance with the recommendations of the Ethics Committee of the Hirosaki University School of Medicine. All precipitants gave written informed consent in accordance with the Declaration of Helsinki. The protocol was approved by the Ethics Committee of the Hirosaki University School of Medicine (No. 2017-026). In this project, a health evaluation was conducted annually for the participants living in the Iwaki area, a suburban area of Hirosaki city in Aomori Prefecture, which is comprised mainly of farmland, and is located in northern Japan (16, 17). For the Iwaki study 2017, there were 1,073 volunteers (*n* = 1,073), of whom the following were excluded: five with incomplete clinical data, 40 with no P-IES measurement, two with no validated P-IES results, and 81 with diabetes diagnosed by the 2010 Japan Diabetes Society criteria (IFG: fasting blood glucose levels 110–125 mg/dl; diabetes: fasting blood glucose levels ≥126 mg/dL or HbA1c levels ≥6.5%) (18) (Figure 1). Those receiving medication for diabetes with a normal range of blood glucose were also defined as having diabetes. We also excluded two individuals with fasting blood glucose levels lower than 63 mg/dl. After these exclusions, 894 normoglycemic individuals (352 men, 542 women) aged 53.0 ± 0.51 years were examined (Figure 1). Fifty-five precipitants with IFG (29 men, 26 women) aged 66.1 ± 1.4 years (*p* < 0.001 vs. normoglycemic participants) were separately examined for the comparison of P-IES with the non-IFG participants.

### Clinical Profiles

Blood samples were collected under fasting conditions in the morning from peripheral veins while in a supine position. The following clinical measurements were recorded: height, body weight, body mass index (BMI), waist circumference, percent body fat (fat), fasting blood glucose (FBG), Achilles tendon reflex (ATR), fasting serum insulin levels (F-IRI), HbA1c, systolic blood pressure (sBP), diastolic BP (dBP), total serum levels of total cholesterol (Tc), triglyceride (Tg), high-density lipoprotein-cholesterol (HDL-c), low-density lipoprotein-cholesterol (LDL-c), interleukin-6 (IL-6), high-sensitivity C-reactive-protein (Hs-CRP), and adiponectin. Adipose tissue volume was measured by the bioelectricity impedance method using a Tanita MC-190 body composition analyzer (Tanita Corp., Tokyo, Japan). ATR was scored based on two titer systems: score 0, areflexia/hyporeflexia; score 1, normal/hyperreflexia. HbA1c (%) was expressed as the National Glycohemoglobin Standardization Program value. To explore the involvement of HbA1c in altered P-IES, we divided the control group into three groups (L-A1c <5.4%, M-A1c 5.4–5.8%, and H-A1c >5.8%) and examined their relationships with clinical measurements and P-IES. The upper limit for the HbA1c in H-A1c was <6.5. Indices of insulin resistance and secretion were assessed by employing a homeostasis model using FBG and insulin levels (HOMA-IR and HOMA-ß), respectively. None of the participants was having diagnosed as type 1 diabetes or inherited diseases that affected HbA1c values. Hypertension was defined as blood pressure ≥ 140/90 mm Hg or a history of treatment for hypertension. Hyperlipidemia was defined as Tc ≥ 220 mg/dL, Tg ≥ 150 mg/dL or a prescription for hyperlipidemia. Alcohol intake (current or non-drinker), smoking habits (current or non-smoker), packs per year and subjective neuropathic foot symptoms (pricking, burning, and aching pains) were determined from questionnaires.

### P-IES Measurement

For nociceptive stimulation, an IES method was adopted using a disposable concentric bipolar needle electrode (NM-983W; Nihon Kohden Corp., Tokyo, Japan) which was connected to a specific stimulator for cutaneous Aδ and C fibers as previously described (PNS-7000; Nihon Kohden) (15).

The stimulator was composed of an outer ring anode (1.2 mm diameter), and the cathode of an inner needle that protruded 0.1 mm from the level of the outer ring. We placed the IES electrode onto the skin of the instep (over the extensor digitorum brevis) and then delivered weak continuous electrical stimulations. If the keratinized layer of the skin was too thick to interrupt the electronic stimulation, the electrode was moved elsewhere on the same foot to where there was seemingly no thick layer. Stimulation intensity was decreased by 0.05 mA stepwise from 0.2 mA until the participants reported a pricking sensation. P-IES was defined as the minimum intensity at which the participants felt a pricking sensation in more than two trials.

### Statistical Methods

Values of the clinical measures are expressed as means ± SEM. The statistical significance of the difference in values between two groups (parametric) and case-control associations among groups (non-parametric) were assessed using an analysis of variance with a *post-hoc* test followed by Bonferroni's corrections and χ^2^ tests, respectively. Correlations between P-IES values and clinical parameters, including age (years), height (cm), BMI, fat (%), waist circumference (cm), decreased ATR, FBG (mg/dl), HbA1c (%), F-IRI (uM/ml), HOMA-β, HOMA-IR, sBP, dBP, Tc (mg/dl), LDL-c (mg/dl), HDL-c (mg/dl), IL-6 (pg/ml), Hs-CRP (mg/dl), adiponectin (μg/ml), and prevalence of hypertension (%), dyslipidemia (%), alcohol habit (%), smoking habit (%), packs per year, and subjective symptoms were assessed by linear regression analyses. For the statistical analyses, HbA1c was log-transformed (log10) to approximate a normal distribution. The risk of higher serum HbA1c with increased P-IES indices was calculated by multiple logistic regression analysis with an adjustment for factors found to be associated with P-IES indices by a univariate regression analysis and the variables that were potentially to be cofounders for DPN from a previous study (19). For the calculation of odds ratios, SFN was designated as 0.25 mA and higher. A value of *p* < 0.05 was regarded as statistically significant. All analyses were performed using Jmp version 10.0.4 and StatView version 5.0.1 (SAS Institute Inc., Cary, NC. USA).

## Results

### Clinical Profiles of the Study Participants

Clinical profiles of the participants are shown in Table 1. The mean age was 51.9 ± 0.8 years for men and 53.7 ± 0.7 years for women. Serum tryglyceride was higher and HDL-c and adiponectin levels were lower in the men (tryglyceride: 121.1 ± 4.8 vs. 78.9 ± 1.8 mg/dL; HDL-c: 60.0 ± 0.9 vs. 70.8 ± 0.7 mg/dL; and adiponectin: 8.6 ± 0.1 vs. 13.6 ± 0.3 mg/ml). The prevalence of hypertension was comparable between men and women (26.1 and 22.5%), likewise that of dyslipidemia (8.9 and 10.1%). Men were more likely to drink alcohol and to be current smokers than women (69.1 vs. 33.0% for alcohol, and 37.1 vs. 23.1% for current smoking, respectively). The prevalence of subjective neuropathic symptoms (1.42 and 1.85%) and decreased ATR (21.59 and 14.94%), were comparable between men and women, respectively.

**Table 1:** Clinical profiles of examined subjects.

	**Men**	**Women**	***p***
n	352	542	–
Age (years)	51.86 ± 0.81	53.71 ± 0.65	0.075
Height (cm)	169.08 ± 0.36	156.12 ± 0.27	<0.0001
Body weight (kg)	67.90 ± 0.58	53.63 ± 0.36	<0.0001
BMI (kg/m^2^)	23.73 ± 0.18	22.03 ± 015	<0.0001
Fat (%)	23.73 ± 0.18	22.03 ± 0.15	<0.0001
Waist circumference (cm)	88.11 ± 0.49	80.98 ± 0.40	<0.0001
FBG (mg/dl)	92.40 ± 0.43	89.29 ± 0.35	<0.0001
HbA1c (%)	5.53 ± 0.02	5.56 ± 0.01	0.2082
F-IRI (μU/ml)	4.88 ± 00.17	4.92 ± 0.09	0.8405
HOMA-β	61.88 ± 2.27	70.54 ± 0.14	0.0006
HOMA-IR	1.13 ± 0.04	1.10 ± 0.02	0.5657
sBP (mmHg)	124.13 ± 0.93	119.42 ± 0.73	<0.0001
dBP (mmHg)	74.42 ± 0.63	69.23 ± 0.46	<0.0001
Tc (mg/dl)	203.42 ± 1.78	209.35 ± 1.48	0.0111
Tg (mg/dl)	121.06 ± 4.79	78.94 ± 1.83	<0.0001
HDL-c (mg/dl)	59.95 ± 0.89	70.83 ± 0.70	<0.0001
LDL-c (mg/dl)	114.05 ± 1.52	116.05 ± 1.23	0.3068
IL-6 (pg/ml)	1.64 ± 0.21	1.40 ± 0.20	0.4156
Hs CRP (mg/dl)	0.06 ± 0.01	0.05 ± 0.01	0.0762
Adiponectin (μg/ml)	8.58 ± 0.02	13.63 ± 0.28	<0.0001
Hypertension: n (%)	26.14	22.51	0.2454
Dyslipidemia: n (%)	8.86	10.07	0.6265
Alcohol habit: n (%)	69.14	32.97	<0.0001
Smoking habit: n (%)	37.14	23.08	<0.0001
Pack a year	13.66 ± 1.21	2.52 ± 0.28	<0.0001
Subjective symptoms: n (%)	1.42	1.85	0.8287
Decreased ATR: n (%)	21.59	14.94	0.2745
P-IES	0.156 ± 0.01	0.14 ± 0.01	0.1635

*BMI, Body mass index; FBG, Fasting plasma glucose; F-IRI, Fasting serum insulin; HOMA-β, homeo static model assessment β cell function; HOMA-IR, homeo static model assessment insulin resistance; sBP, systolic blood pressure; dBP, diastolic blood pressure; Tc, Total cholesterol; Tg, Triglyceride; HDL-c, High density lipoprotein cholesterol; LDL-c, low density lipoprotein cholesterol; IL-6, interleukin-6; Hs CRP, High sensitivity C-reactive protein; ATR, Achilles tendon reflex; P-IES, Pain threshold of intraepidermal electrical stimulation*.

### Age-Related P-IES Changes

**Figure 2:**
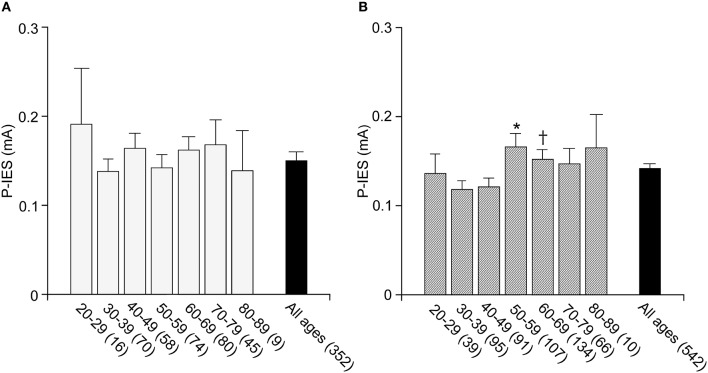
P-IES in male and female. P-IES is consistent during each decade of life in men **(A)**. In contrast, women of 50–59 and 60–69 years old showed a significant increase in P-IES compared to 30–39 years old **(B)**. Mean ± SEM, *p < 0.01 vs. 30–39, *p* < 0.05 vs. 40–49, ^†^*p* < 0.05 vs. 30–39.

To date, no standard value for P-IES has been determined in a general Japanese population. Further, the P-IES changes due to aging and sex differences have not been evaluated. Therefore, we examined those measures in a general Japanese population from the Iwaki study. The number of examined cases for each decade of age in men and women is listed in Table 2. The men's average P-IES (mA) for all ages was 0.15 ± 0.01, with a 95% confidence interval (CI) of was “0.14” to “0.17.” That of the women was comparable, with a value of 0.14 ± 0.01 and a 95% CI of “0.13” to “0.15” (Figure 2). The average total value was 0.15 ± 0.01 with a 95% CI of “0.14” to “0.16.” The 95th percentile of the total population is 0.16. Therefore, more than 0.15 is regarded as an abnormal P-IES in the total population. Age did not influence P-IES in men, whereas in women aged in their 50s (0.17 ± 0.02) and 60s (0.16 ± 0.01), the values were significantly higher than those in their 30s (0.12 ± 0.01) (*p* < 0.01 and *p* < 0.05, respectively). The values in women in their 50s were also significantly higher than those in their 40s (*p* < 0.05). There was a trend toward a high P-IES in women in their 70s and 80s compared with the values in their 30s and 40s (*p* = 0.06, 40s vs. 70s).

**Table 2:** The examined number of cases in each decade of age.

**Decade of age (years)**	**Male**	**Female**	**Total**
20–29	16	39	55
30–39	70	95	165
40–49	58	91	149
50–59	74	107	181
60–69	80	134	214
70–79	45	66	111
80-89	9	10	19
Total	352	542	894

### Correlation Between P-IES Indices and Serum HbA1c

A univariate regression analysis revealed a close correlation between the P-IES values and clinical measurements such as age, BMI, waist circumference, sBP, presence of hypertension, decreased ATR, FBG, and HbA1c (Table 3). The correlation between P-IES and HbA1c remained significant after adjustment for multiple factors (age, BMI, waist circumference, sBP, hypertension, decreased ATR, and FBG) (ß = 0.08; *p* = 0.046). These correlations remained significant after adjustment for gender sex and age (HbA1c: ß = 0.0784; *p* = 0.032) (Table 4).

**Table 3:** Clinical factors correlated with P-IES indices.

	**Univariate**		**Multivariate**	
**Characteristics**	**β**	***p***	**β**	***p***
Sex (men/women)	0.0488	0.1446	-	-
Age (years)	0.47610	0.001	0.07396	0.0832
Height (cm)	−0.02268	0.4978	-	-
Body weight (kg)	0.059134	0.0769	-	-
BMI (kg/m^2^)	0.07997	0.0168	–0.0188	0.8035
Fat (%)	0.030964	0.3554	-	-
Waist circumference (cm)	0.09508	0.0044	0.06913	0.3697
FBG (mg/dl)	0.09668	0.0038	0.01709	0.6654
HbA1c(%)	0.1202	0.0003	0.07657	0.0459
F-IRI (μU/ml)	0.026419	0.4294	-	-
HOMA-β	−0.02683	0.4228	-	-
HOMA-IR	0.039726	0.2346	-	-
sBP (mmHg)	0.091228	0.0063	0.02575	0.5074
dBP (mmHg)	0.03714	0.2665	-	-
Tc (mg/dl)	0.001451	0.9654	-	-
Tg (mg/dl)	0.044434	0.1836	-	-
HDL-c (mg/dl)	−0.0549	0.1004	-	-
LDL-c (mg/dl)	0.022807	0.4951	-	-
IL-6 (pg/ml)	0.029864	0.3717	-	-
Hs CRP (mg/dl)	0.028409	0.3954	-	-
Adiponectin (μg/ml)	−0.02645	0.4288	-	-
Hypertension: n (%)	0.068749	0.0399	–0.03003	0.3843
Dyslipidemia: n (%)	0.023986	0.4731	-	-
Alcohol habit: n (%)	−0.01053	0.7528	-	-
Smoking habit: n (%)	0.295599	0.1128	-	-
Pack a year	−0.03481	0.2976	-	-
Subjective symptoms	0.019313	0.5641		
Decreased ATR	−0.07009	0.0362	–0.02693	0.4338

*BMI, Body mass index; FBG, Fasting plasma glucose; F-IRI, Fasting serum insulin; HOMA-β, homeo static model assessment β cell function; HOMA-IR, homeo static model assessment insulin resistance; sBP, systolic blood pressure; dBP, diastolic blood pressure; Tc, Total cholesterol; Tg, Triglyceride; HDL-c, High density lipoprotein cholesterol; LDL-c, low density lipoprotein cholesterol; IL-6, interleukin-6; Hs CRP, High sensitivity C-reactive protein; and ATR, Achilles tendon reflex*.

**Table 4:** Correlation of HbA1c with P-IES indices.

	**Univariate**		**Age and gender adjusted**		**Multivariate**	
	**β**	***p***	**β**	***p***	**β**	***p***
HbA1c (%)	0.1202	0.0003	0.0784	0.0320	0.0751	0.0486

### Comparison of Clinical Measurements Among Stratified Groups of Graded HbA1c and IFG and the Risk of Elevated P-IES

To explore the implication of HbA1c in altered P-IES, the IFG group was also included for the comparison. The prevalence of abnormal P-IES, which is more than 0.15, was 18 out of 51 cases in IFG (35.3%).

**Figure 3:**
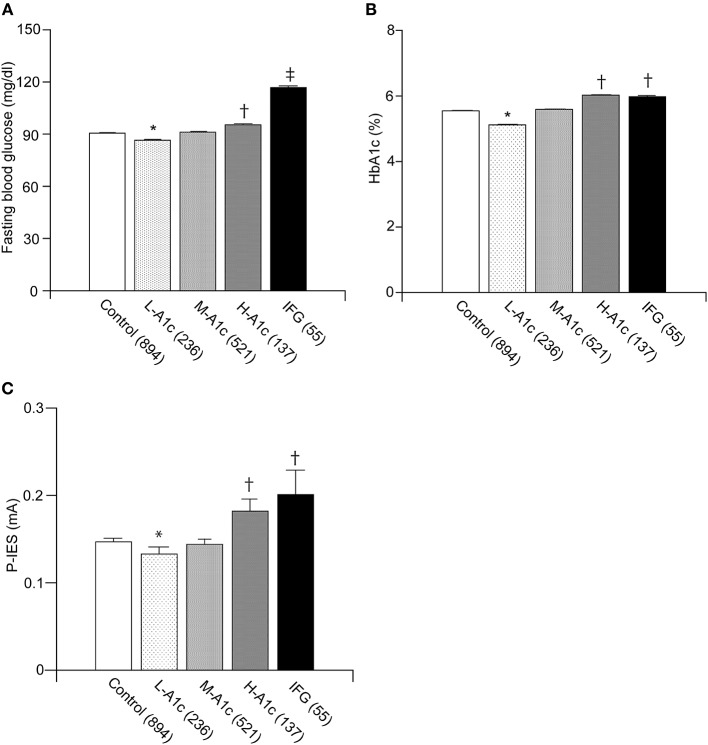
Comparison between IFG and each group of graded HbA1c in normoglycemic individuals. Fasting blood glucose values were in parallel with L-A1c, M-A1c, and H-A1c **(A)**. HbA1c and P-IES of IFG were comparable to those of H-A1c **(B,C)**. L-A1c, HbA1c low; M-A1c, HbA1c moderate; H-A1c, HbA1c high. Mean ± SEM, *p < 0.01 vs. L-A1c, ^†^*p* < 0.01 vs. Control, L-A1c, M-A1c, ^‡^*p* < 0.01 vs. H-A1c.

The average P-IES values for L-A1c, M-A1c, H-A1c, and IFG were 0.13 ± 0.01, 0.14 ± 0.01, 0.18 ± 0.01, and 0.20 ± 0.03, respectively. In the IFG groups, the FBG and P-IES indices were increased stepwise in parallel with HbA1c values (p < 0.01) (Figures 3A–C). FBG was significantly increased in IFG compared with H-A1c (p < 0.001) (Figure 3A). FBG, HbA1c, and P-IES indices in IFG were all significantly increased compared with those of the control group and L-A1c values (p < 0.01) (Figures 3A–C).

**Figure 4:**
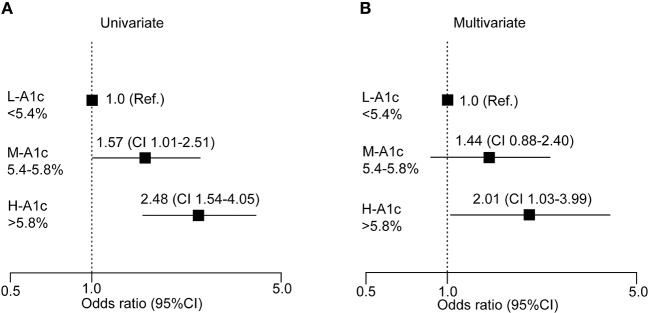
Logistic analysis of clinical measures at risk for increased P-IES. Odds ratio with 95% confidence interval (CI) for HbA1c **(A,B)** are shown. Multiple factors are age, BMI, waist circumference, decreased ATR, FBS, sBP, and hypertension. Ref: reference. L-A1c: HbA1c low, M-A1c: HbA1c moderate, H-A1c: HbA1c high.

We further conducted a logistic regression analysis of graded levels of HbA1c and the risk of elevated P-IES (Figure 4A). If small fiber dysfunction was designated as 0.20 mA or more, high serum HbA1c (>5.8%) was a significant risk factor for increased P-IES. The risk remained significant after adjustment for multiple factors (age, BMI, waist circumference, decreased ATR, FBG, sBP, and hypertension) (Figure 4B).

### Correlation Between P-IES Indices and Other Peripheral Nerve Tests

We evaluated the correlation between P-IES and decreased ATR and the presence of subjective symptoms. Although the prevalence of hyporeflexia gradually increased in parallel with HbA1c level, a significant difference was only observed between H-A1c and L- A1c (*p* < 0.05) (Table 5). The prevalence of hyporeflexia in IFG was significantly higher than that of M-A1c, whereas the prevalence in IFG was comparable to with that of H-A1c (Table 5). In contrast, there was no difference in subjective symptoms among control subjects according to HbA1c concentration (Table 6). The prevalence of subjective symptoms in IFG was the highest among all groups (L-A1c vs. H-A1c, *p* < 0.05; Control vs. M-A1c, *p* < 0.01) (Table 6).

**Table 5:** Correlation of normal high HbA1c and IFG with decreased ATR.

	**ATR**		
	**Normal**	**Weak**	**Total**
Control	82.4% (737)	17.6% (157)	100% (894)
L-A1c	89.4% (211)	10.6% (25)	100% (236)
M-A1c	81.2% (423)	18.8% (98)	100% (521)
H-A1c	75.2% (103)	24.8% (34)^*^	100% (137)
IFG	64.7% (33)	35.3% (18)^†^	100% (51)

* p < 0.05 vs. L-A1c,

† *p < 0.01 vs. L-A1c and Control, p < 0.05 vs. M-A1c. ATR, Achilles tendon reflex; IFG, impaired fasting glucose; and ATR, Achilles tendon reflex*.

**Table 6:** Correlation of HbA1c and IFG with subjective neuropathic symptoms.

	**Subjective symptoms**		
	**-**	**+**	**Total**
Control	98.5% (881)	1.5(13)	100% (894)
L-A1c	98.3% (232)	1.7% (4)	100% (236)
M-A1c	98.8% (515)	1.2% (6)	100% (521)
H-A1c	97.8% (134)	2.2% (3)	100% (137)
IFG	90.2% (46)	9.8% (5)^*^	100% (51)

* *p < 0.05 vs. L-A1c and H-A1c, p < 0.01 vs. Control and M-A1c. IFG, impaired fasting glucose*.

## Discussion

In this study, we established a normal P-IES reference value in a healthy non-diabetic Japanese population. Average values in the controls without diabetes were consistent at ~0.15 mA in all decades of life in men and 0.14 mA in women, although there was slight elevation in women in their 50s and 60s. It was of note that the P-IES was significantly increased in participants with normal high HbA1c levels as well as in participants with IFG participants. A stratified logistic analysis further revealed good correlations between elevated P-IES and higher HbA1c levels. Increased P-IES was repeatedly reported in patients with diabetes (14, 15). Our results thus confirmed the early derangement of pain sensation in participants with IFG as well as in a group of participants with normal high HbA1c levels. In conjunction with the early loss of intraepidermal nerve fibers in participants with prediabetes (7, 20), our study provided evidence that small nerve fibers in the distal leg are commonly affected even in individuals without diabetes as evaluated by HbA1c and FBG.

Our univariate linear regression analysis revealed significant association between elevated P-IES and features of metabolic syndrome such as BMI, waist circumference, FBG, HbA1c, sBP, and hypertension. In contrast, only a high HbA1c level was a risk factor according to the multivariate analysis. HbA1c was a strong predictor of type 2 diabetes in the general population, and individuals with normal high HbA1c values had a higher risk for type 2 diabetes than those with IFG (9, 10). The cases of IGT or diabetes could be included in our cohort because we did not perform oral glucose tolerance test. Nevertheless, our results suggest that normal high HbA1c levels could be a useful index for screening for small nerve fiber dysfunction, which is often detectable as a loss of intraepidermal nerve fibers in subclinical DPN.

SFN often presents as an initially important sign of DPN, particularly in IGT, later progressing to overt DPN. Along these lines, Azmi et al. have reported that individuals with IGT who developed overt diabetes showed a progressive reduction of small fibers in the skin and cornea (7). The elevation of P-IES in H-A1c suggests the presence of either SFN or subclinical DPN. One of characteristics of SFN is the appearance of without subjective symptoms such as neuropathic pain, which could interrupt the assessment of P-IES (4). On the other hand, the prevalence of subjective symptoms in H-A1c was comparable to that in L-A1c and M-A1c. The prevalence of decreased ATR, indicating the defect of large nerve fibers, was significantly pronounced in H-A1c compared with L-A1c and the characteristics of preclinical DPN, and M-A1c, similar to that of IFG. These results might suggest that H-A1c comprises more cases of preclinical asymptomatic DPN than those of SFN. Therefore, P-IES is a useful method to screen for the presence of small fiber dysfunction in individuals with normal high HbA1c which is associated less often with subjective neuropathic symptoms.

The introduction of small electrodes for stimulation of Aδ and C fibers identified an elevated P-IES only in patients with established neuropathy, such as amyloid neuropathy, painful neuropathy and DPN (14, 15, 21, 22). Unfortunately, the quantitative evaluation of small nerve fibers for these diseases was not pathologically confirmed. Our preliminary observation confirmed a good correlation between elevated P-IES and a loss of intraepidermal nerve fibers in patients with diabetes (data not shown). Our results showed that P-IES in individuals with H-A1c was comparable to that of those with IFG. Thus, elevated P-IES could reflect an early disorder of nerve function and an ongoing loss of small nerve fibers occurring even in normal high HbA1c state of subjects.

The mechanism for elevated P-IES in H-A1c in our study is unclear. Interestingly, intermittent high glucose levels evoke greater oxidative stress than mean chronic high glucose (23, 24). The participants with a normal high HbA1c value might exhibit intermittent high glucose levels given they are at a higher risk for onset of type 2 diabetes than those with IFG (9). Oxidative stress is an important risk factor for DPN (1).Thus, the impacts of oxidative stress might affect the small nerve dysfunction in individuals with H-A1c. The loss of mitochondria in epidermal nerve fibers recently demonstrated in patients with early diabetes appeared to be consistent with the implication of oxidative stress and mitochondria in altered P-IES in our participants (25). In addition, advanced glycation endoproducts (AGEs), such as pentosidine, N(epsilon)-(carboxymethyl)lysine, and N(epsilon)-(carboxyethyl)lysine are progressively accumulated in the skin with diabetes patients in parallel with diabetic duration (26, 27). The presence of SFN is associated with a higher accumulation of AGEs in the skins of type 1 diabetic subjects (28). Therefore, accumulation of AGEs in the skin of patients with H-HbA1c could contribute to small fiber dysfunction.

We found an elevation of P-IES in women over 50 years of age, although there was no significant difference in men as they aged. Several reasons can be posited for the sex difference. First, age-related hormone disturbances might be implicated. Several studies have proposed neuroprotective effects of estrogen, however, there was no evidence of SFN (29–31). The imbalance of sex hormone metabolism in middle-aged or older women could increase the vulnerability of nerve fibers. Second, the traditional Japanese custom of sitting up straight might contribute to elevated P-IES in middle-aged or older women by compressing the lower legs.

There are several limitations to this study. First, it is cross-sectional and does not follow the progression or reversal of altered P-IES. Given that it is essential to confirm the implication of glycemic severity in small fiber dysfunction, a meticulous longitudinal study would be required. It should also be noted that the study participants were volunteers in a health promotion study rather than participants in an ordinary health check up. The study participants could, therefore, be more healthy than the general population. We also excluded individuals with diabetes who had only a single measurement of FBG, HbA1c, and a detailed clinical history. Heinza et al. had shown that prediabetes was efficiently detected by the combination tests of FBG (5.6–6.9 mmol/L) and HbA1c (5.7–6.4%) (32). Nevertheless, we could have included small cases of IGT or overt diabetes in the group of normoglycemic individuals, even if those populations could be trivial, given there might be individuals who would only show abnormal glucose tolerance with normal HbA1c and FBG. A glucose tolerance test would be required to confirm the diabetic status of our cohort in the future.

In conclusion, normal high HbA1c levels were significantly associated with elevated P-IES in a general Japanese population without diabetes. Although a longitudinal observation will be required to confirm our results, a logistic analysis revealed that individuals with normal high HbA1c levels might be at risk for elevated P-IES. These results suggest that those with normal high HbA1c levels are likely to develop SFN in diabetes.

## Data Availability Statement

All datasets generated for this study are included in the manuscript.

## Ethics Statement

This study was carried out in accordance with the recommendations of the Ethics Committee of the Hirosaki University School of Medicine with written informed consent from all subjects. All subjects gave written informed consent in accordance with the Declaration of Helsinki. The protocol was approved by the Ethics Committee of the Hirosaki University School of Medicine (No.2017-026).

## Author Contributions

CI conducted the study for the measurement of P-IES, discussed and interpreted the results, and wrote the manuscript. HM designed and conducted the study for the measurement of P-IES, discussed and interpreted the results, and wrote the manuscript. SOs, KT, KKu, WI, GD, CU, SU AI, SOg, MR, and KKo conducted the study for the measurement of P-IES, interpreted and discussed the results. KW conducted the study for the measurement of ATR, interpreted and discussed the results. YI and SY interpreted and discussed the results and wrote the manuscript. SN designed and conducted Iwaki study, interpreted and discussed the results.

### Conflict of Interest

Electrodes for intraepidermal electrical stimulation were supplied by Nihon Kohden Corp., Tokyo, Japan. The authors declare that the research was conducted in the absence of any commercial or financial relationships that could be construed as a potential conflict of interest.
