# Corrigendum: Normal High HbA1c a Risk Factor for Abnormal Pain Threshold in the Japanese Population

**DOI:** 10.3389/fendo.2020.00130

**Published:** 2020-03-10

**Authors:** Chieko Itabashi, Hiroki Mizukami, Sho Osonoi, Kazuhisa Takahashi, Kazuhiro Kudo, Kanichiro Wada, Wataru Inaba, Guo Danyang, Chiaki Uchida, Satoko Umetsu, Akiko Igawa, Saori Ogasawara, Masaki Ryuzaki, Kouji Komeda, Yasuyuki Ishibashi, Soroku Yagihashi, Shigeyuki Nakaji

**Affiliations:** ^1^Department of Pathology and Molecular Medicine, Hirosaki University Graduate School of Medicine, Hirosaki, Japan; ^2^Department of Orthopedic Surgery, Hirosaki University Graduate School of Medicine, Hirosaki, Japan; ^3^Department of Gastrointestinal Surgery, Hirosaki University Graduate School of Medicine, Hirosaki, Japan; ^4^Department of Social Medicine, Hirosaki University Graduate School of Medicine, Hirosaki, Japan

**Keywords:** small fiber dysfunction, diabetic polyneuropathy, HbA1c, painful, small fiber assessment

In the original article, old type of electrodes were incorrectly identified as (NM-990W) instead of (NM-983W). In addition, the average of P-IES in non-diabetic/IFG subjects was incorrect. The correct value is “0.15 ± 0.01.”

A correction has been made in the following places:

The Material and Methods section, subsection P-IES Measurement, paragraph 1:

“For nociceptive stimulation, an IES method was adopted using a disposable concentric bipolar needle electrode (NM-983W; Nihon Kohden Corp., Tokyo, Japan) which was connected to a specific stimulator for cutaneous Aδ and C fibers as previously described (PNS-7000; Nihon Kohden) (15).”

The Abstract, subsection Results:

“P-IES was elevated with increasing of age in women but not in men. Average P-IES (mA) was increased in IFG subjects (*n* = 55, 0.20 ± 0.03) compared with normoglycemic/non-IFG individuals (*n* = 894, 0.15 ± 0.01) (*p* < 0.01). It was comparable between IFG and a group of normal high HbA1c (5.9–6.4%). Univariate linear regression analyses showed no influence of sex, triglyceride, or cholesterol on the value of P-IES. In contrast, there were significant correlations between P-IES and serum HbA1c level (ß = 0.120, *p* < 0.001) Adjustments for the multiple clinical measurements confirmed positive correlation of P-IES with HbA1c (ß = 0.077, *p* = 0.046).”

The authors apologize for this error and state that this does not change the scientific conclusions of the article in any way. The original article has been updated.

